# Supplementary material for: Expression of EMT-Related Genes in Hybrid E/M Colorectal Cancer Cells Determines Fibroblast Activation and Collagen Remodeling
Source: Int J Mol Sci. 2020 Oct 30;21(21):8119. doi: 10.3390/ijms21218119 (PMC7662237; doi:10.3390/ijms21218119)
Supplement: Supplementary file 1 [file ijms-21-08119-s001.zip › Collagen_suppl_new.doc]

Supplementary Materials

Expression of EMT related genes in hybrid E/M colorectal cancer cells determines fibroblast activation and collagen remodeling

Irina Druzhkova, Marina Shirmanova, Nadezhda Ignatova, Varvara Dudenkova, Maria Lukina, Elena Zagaynova, Dina Safina, Sergey Kostrov, Dmitry Didych, Alexey Kuzmich, George Sharonov, Olga Rakitina, Irina Alekseenko, Eugene Sverdlov

**Figure S1.** Comparison of transcriptomes for HT29 and SW480 colorectal cancer cell lines and subsequent Gene Ontology-based functional enrichment analysis of DEGs. (A) The revealing of SW480 and HT29 DEGs by comparing RNA-seq data, all in triplicate, using DESeq2. Volcano plot showing the distribution of gene expression fold changes (log2(FC) and adjusted p-values (-log10(p-adj) for all genes whose expression is not lower than 5 TPM in at least one of the compared cell lines. Red genes - DEGs more expressed in HT29 (p-adj < 0.05, log2(FC) ≤ -1), blue – DEGs more expressed in SW480 (p-adj < 0.05, log2(FC) ≥ 1). Point-size reflects the gene expression level in TPM. (B) Gene Ontology functional enrichment analysis of the DEGs in SW480 и HT29 using DAVID. REVIGO was used for visualization Gene Ontology Biological Processes with p-adj value < 0.05. Orange color highlights terms associated with migration processes, green color – terms associated with adhesion processes. The size of the circles is proportional -log10(p-adj).

**Figure S2.** Expression of the genes, related to collagen metabolism and its regulation in normal fibroblasts and CAFs, isolated from the patient’s colorectal tumors. A – Box-plots represent the difference of collagen-related gene expression between NFs and CAFs. The distributions of log2(FC) value for significantly expressed collagen-related genes (p-adj < 0.05; TPM ≥ 5) in 3 gene categories (B+PR, CFO+EA, and C+NR) are shown, that is, the expression of the gene in CAFs divided by the expression of the gene in NFs. Thus, gene, expression of which is greater in CAFs, has log2(FC) > 0, and gene, expression of which is greater in NFs, has log2(FC) < 0. Twenty-fifth, fiftieth, and seventy-fifth percentiles are used; whiskers show minimal and maximal log2(FC)-values. White boxes represent the difference in collagen-related gene expression between NFs and CAF1; grey boxes represent the difference in collagen-related gene expression between NFs and CAF2. Red line indicates log2(FC) = 0, that is, no change in gene expression. The number of the genes (n) used for each box is indicated on the right side of the chart. B – Histogram showing the expression NF-specific (red gene name) and CAF1-specific (blue gene name) collagen-related genes (p-adj < 0.05; TPM ≥ 5;│log2(FC)│≥ 1). Red bars represent the expression of genes in NF, blue bars represent the expression of genes in CAF1. C – Histogram showing the expression of NF-specific (red gene name) and CAF2-specific (blue gene name) collagen-related genes (p-adj < 0.05; TPM ≥ 5;│log2(FC)│≥ 1). B, C - Mean values (n=3) are shown in TPM, error bars represent standard deviation (n=3).

**Figure S3.** Differential expression of the collagen-related genes in co-cultivated cancer cells and fibroblasts. (A) Histogram showing the expression of collagen-related DEGs (p-adj < 0.05; TPM ≥ 5;│log2(FC)│≥ 1) in HT29 (red bars) and SW480 (blue bars) after co-cultivation. Red gene names represent genes, up-regulated in HT29^NF^ compared to SW480^NF^, blue gene names represent genes, up-regulated in SW480^NF^ compared to HT29^NF^. Mean values (n=3) are shown, error bars represent standard deviation (n=3). Up and down arrows indicate genes, that are up- or down-regulated in HT29 (red arrows) or in SW480 (blue arrows) cells after co-cultivation. Three gene categories are shown: Collagen biosynthesis and positive regulation (B+PR), Collagen fibril organization and ECM aggregation (CFO), and Collagen catabolism and negative regulation (C+NR). (B) Histogram showing the expression of collagen-related DEGs (p-adj < 0.05; TPM ≥ 5;│log2(FC)│≥ 1) in NFs after co-cultivation with HT29 (red bars) or SW480 (blue bars). Red gene names represent genes, up-regulated in NF^HT29^ compared to NF^SW480^, blue gene names represent genes, up-regulated in NF^SW480^ compared to NF^HT29^. Mean values (n=3) are shown, error bars represent the standard deviation (n=3). Up and down arrows indicate genes, that are up- or down-regulated in NFs after co-cultivation with HT29 (red arrows) or with SW480 (blue arrows).

**Table S1.** Clinicopathologic characteristics of surgical specimens used for CAFs isolation.

| **n/n** | **Tumor Site** | **Tumor Type** | **Tumor Stage** | **Differentiation** | **Patient gender** | **Patient age** |
| --- | --- | --- | --- | --- | --- | --- |
| 1 (CAF1) | Primary, sigmoid | Adenocarcinoma | T3N1M0 (stage III) | Moderate | female | 61 |
| 2 (CAF2) | Primary, rectum | Adenocarcinoma | T3N1M1 (stage IV) | Moderate | male | 68 |
| 3 (CAF) | Primary, rectum | Adenocarcinoma | T4N1M1 (stage IV) | Moderate | female | 58 |

**Table S2.** List of epithelial marker genes.

| **Official gene symbol** | **Gene name** | **Gene category** | **Source** |
| --- | --- | --- | --- |
| CDH1 | cadherin 1 | Cell adhesion protein | [14,15,17] |
| COL4A1 | collagen type IV alpha 1 chain | ECM component (basal membrane) | [14,17] |
| DSC1 | desmocollin 1 | Cell adhesion protein (desmosome component) | [14] |
| DSC2 | desmocollin 2 | Cell adhesion protein (desmosome component) | [14] |
| DSC3 | desmocollin 3 | Cell adhesion protein (desmosome component) | [14] |
| DSG1 | desmoglein 1 | Cell adhesion protein (desmosome component) | [14] |
| DSG2 | desmoglein 2 | Cell adhesion protein (desmosome component) | [14] |
| DSG3 | desmoglein 3 | Cell adhesion protein (desmosome component) | [14] |
| DSG4 | desmoglein 4 | Cell adhesion protein (desmosome component) | [14] |
| DSP | desmoplakin | Cell adhesion protein (desmosome component) | [17] |
| EPCAM | epithelial cell adhesion molecule | Cell adhesion protein | [16] |
| ITGB4 | integrin subunit beta 4 | Cell adhesion protein | [14] |
| KLF5 | Kruppel like factor 5 | Transcription factor | [15] |
| KRT18 | keratin 18 | Cytoskeleton protein | [14,17] |
| KRT19 | keratin 19 | Cytoskeleton protein | [14,17] |
| KRT5 | keratin 5 | Cytoskeleton protein | [14,17] |
| LAMA1 | laminin subunit alpha 1 | ECM component (basal membrane) | [14,17] |
| LAMA2 | laminin subunit alpha 2 | ECM component (basal membrane) | [14,17] |
| LAMA3 | laminin subunit alpha 3 | ECM component (basal membrane) | [14,17] |
| LAMA4 | laminin subunit alpha 4 | ECM component (basal membrane) | [14,17] |
| LAMA5 | laminin subunit alpha 5 | ECM component (basal membrane) | [14,17] |
| MMP7 | matrix metallopeptidase 7 | Protease (matrix metallopeptidase) | [14] |
| MUC1 | mucin 1, cell surface associated | Cell adhesion protein, transcription factor | [17] |
| NID1 | nidogen 1 | ECM component (basal membrane) | [17] |
| OCLN | occludin | Cell adhesion protein (tight junction component) | [17] |
| TJP1 | tight junction protein 1 | Cell adhesion protein (tight junction component) | [17] |

**Table S3.** List of mesenchymal marker genes.

| **Official gene symbol** | **Gene name** | **Gene category** | **Source** |
| --- | --- | --- | --- |
| ACTA2 | actin, alpha 2, smooth muscle, aorta | Cytoskeleton protein | [17] |
| CDH11 | cadherin 11 | Cell adhesion protein | [14,17] |
| CDH2 | cadherin 2 | Cell adhesion protein | [14,17] |
| COL1A1 | collagen type I alpha 1 chain | ECM component in connective tissues | [14] |
| COL3A1 | collagen type III alpha 1 chain | ECM component in connective tissues | [14] |
| COL5A1 | collagen type V alpha 1 chain | ECM component in connective tissues | [14] |
| CTNNB1 | catenin beta 1 | Cytoskeleton protein | [17] |
| DDR2 | discoidin domain receptor tyrosine kinase 2 | Receptor tyrosine kinase | [17] |
| FN1 | fibronectin 1 | ECM component | [14,15,17] |
| FOXC2 | forkhead box C2 | Transcription factor | [17] |
| GSC | goosecoid homeobox | Transcription factor | [17] |
| ITGA5 | integrin subunit alpha 5 | Integrin receptor part | [14] |
| ITGAV | integrin subunit alpha V | Integrin receptor part | [14] |
| ITGB1 | integrin subunit beta 1 | Integrin receptor part | [14] |
| ITGB3 | integrin subunit beta 3 | Integrin receptor part | [14] |
| ITGB6 | integrin subunit beta 6 | Integrin receptor part | [17] |
| KRT8 | keratin 8 | Cytoskeleton protein | [17] |
| LEF1 | lymphoid enhancer binding factor 1 | Transcription factor | [17] |
| MMP11 | matrix metallopeptidase 11 | Protease (matrix metallopeptidase) | [14] |
| MMP14 | matrix metallopeptidase 14 | Protease (matrix metallopeptidase) | [14] |
| MMP2 | matrix metallopeptidase 2 | Protease (matrix metallopeptidase) | [17] |
| MMP3 | matrix metallopeptidase 3 | Protease (matrix metallopeptidase) | [14,17] |
| MMP9 | matrix metallopeptidase 9 | Protease (matrix metallopeptidase) | [17] |
| S100A4 | S100 calcium binding protein A4 | Activator of NF-kappaB signaling | [14,17] |
| SDC1 | syndecan 1 | Cell adhesion protein | [17] |
| SERPINH1 | serpin family H member 1 | Protease inhibitor | [17] |
| SMAD2 | SMAD family member 2 | Transcription factor | [17] |
| SMAD3 | SMAD family member 3 | Transcription factor | [17] |
| SNAI1 | snail family transcriptional repressor 1 | Transcription factor | [17] |
| SNAI2 | snail family transcriptional repressor 2 | Transcription factor | [17] |
| TCF3 | transcription factor 3 | Transcription factor | [17] |
| TCF4 | transcription factor 4 | Transcription factor | [17] |
| TNC | tenascin C | ECM protein | [14] |
| TWIST1 | twist family bHLH transcription factor 1 | Transcription factor | [17] |
| VIM | vimentin | Cytoskeleton protein | [14,17] |
| ZEB1 | zinc finger E-box binding homeobox 1 | Transcription factor | [17] |
| ZEB2 | zinc finger E-box binding homeobox 2 | Transcription factor | [17] |

**Table S4.** List of CAF marker genes.

| **Official gene symbol** | **Gene name** | **Gene category** | **Source** |
| --- | --- | --- | --- |
| ACTA2 | actin, alpha 2, smooth muscle, aorta | Cytoskeleton protein | [19-22,24] |
| ASPN | asporin | ECM component | [18] |
| C5AR2 | complement component 5a receptor 2 | Complement system component | [22] |
| CCL5 | C-C motif chemokine ligand 5 | Chemokine | [25] |
| CSPG4 | chondroitin sulfate proteoglycan 4 | Proteoglycan (integral) | [19] |
| CXCL10 | C-X-C motif chemokine ligand 10 | Chemokine | [25] |
| CXCL12 | C-X-C motif chemokine ligand 12 | Chemokine | [20] |
| DES | desmin | Cytoskeleton protein | [19,20] |
| FAP | fibroblast activation protein alpha | Protease (integral membrane gelatinase) | [19-22,24] |
| IL6 | interleukin 6 | Interleukin | [25] |
| MME | membrane metalloendopeptidase | Protease (metallopeptidase) | [21,22] |
| PDGFRA | platelet derived growth factor receptor alpha | Cellular signaling-involved protein (Ras signaling) | [19,21] |
| PDGFRB | platelet derived growth factor receptor beta | Cellular signaling-involved protein (Ras signaling) | [19-22] |
| PDPN | podoplanin | Transmembrane transporter | [21] |
| POSTN | periostin | ECM component | [19] |
| PRRX1 | paired related homeobox 1 | Transcription factor | [23] |
| S100A4 | S100 calcium binding protein A4 | Cellular signaling-involved protein (Activator of NF-kappaB signaling) | [19-22] |
| SNAI2 | snail family transcriptional repressor 2 | Transcription factor | [21] |
| STC1 | stanniocalcin 1 | Hormone | [18] |
| THY1 | Thy-1 cell surface antigen | Cell adhesion protein | [24] |
| TNC | tenascin C | ECM component | [19,23] |
| TWIST1 | twist family bHLH transcription factor 1 | Transcription factor | [21,23] |
| VIM | vimentin | Cytoskeleton protein | [19,20] |
| ZEB1 | zinc finger E-box binding homeobox 1 | Transcription factor | [21] |

**Table S5.** Quantitative parameters of SHG signal in monocultures of colorectal cancer cells in a collagen-based model. Mean±SEM.

| **Parameter** | **Cell culture** | **Days of cultivation** | | | | |
| --- | --- | --- | --- | --- | --- | --- |
|  |  | **1** | **2** | **3** | **7** | **9** |
| **Mean (a.u.)** | **HT29** | 2862±323,8 | 1497,2±476,0 | 1804,7±379,4 | 1432,2±139 | 835,7±400,6 |
|  | **HCT116** | 2168,8±245,7 | 1771,2±335,6 | 3455±734,1 | 1562,0±318,7 | 2241,2±143,8 |
|  | **SW480** | 1667,2±317,1 | 1825,4±187,9 | 2984,7±865,6 | 2008±700,2 | 3484,2±836,3 |
| **Median (a.u.)** | **HT29** | 2691,8±329,3 | 1351±465,1 | 1662±377,2 | 1302,5±147,9 | 701,7±358,9 |
|  | **HCT116** | 1992,4±239,2 | 1579,8±326,1 | 3227,1±724,4 | 1423,8±334 | 2087±150,6 |
|  | **SW480** | 1447,8±257,2 | 1662,5±191,4 | 1907,1±650,7 | 1290,3±390,3 | 2802,7±683,6 |
| **Density (a.u.)** | **HT29** | 4159,7±470,6 | 2176±691,6 | 2622,9±551,4 | 1997,5±193,8 | 1165,5±558,8 |
|  | **HCT116** | 3376,3±361,4 | 2574,3±487,8 | 5021,4±1066,9 | 2178,6±444,6 | 3125,9±200,5 |
|  | **SW480** | 2423,2±460,8 | 2653,1±273,2 | 4221,2±1185,1 | 2800,6±976,5 | 4808±1207,9 |
| **Skew (a.u.)** | **HT29** | 0,93±0,18 | 1,65±0,71 | 1,19±0,20 | 1,35±0,32 | 1,88±0,78 |
|  | **HCT116** | 1,42±0,30 | 1,67±0,43 | 1,31±0,62 | 1,03±0,29 | 0,86±0,012 |
|  | **SW480** | 2,49±0,57 | 1,38±0,40 | 3,07±1,00 | 3,61±0,97 | 2,33±0,98 |
| **Kurtosis (a.u.)** | **HT29** | 2,21±1,35 | 7,82±6,39 | 3,66±1,30 | 5,34±2,76 | 13,25±6,47 |
|  | **HCT116** | 6,13±3,46 | 6,66±3,00 | 4,81±2,04 | 1,91±1,21 | 1,38±0,47 |
|  | **SW480** | 15,46±6,11 | 4,89±2,75 | 16,11±8,04 | 23,15±12,27 | 10,45±9,17 |
| **Non-**  **Uniformity (a.u.)** | **HT29** | 0,474±0,033 | 0,645±0,125 | 0,558±0,049 | 0,611±0,048 | 0,822±0,072 |
|  | **HCT116** | 0,544±0,03 | 0,627±0,074 | 0,478±0,069 | 0,599±0,085 | 0,506±0,025 |
|  | **SW480** | 0,697±0,086 | 0,58±0,05 | 1,118±0,256 | 1,174±0,122 | 0,755±0,187 |
| **Energy (a.u.)** | **HT29** | 4196321±665895 | 2107794±698953 | 2543056±639509 | 1944381±172726 | 1188537±857458 |
|  | **HCT116** | 3301862±340990 | 2845262±717550 | 6325455±1683092 | 2076919±396982 | 3154630±200227 |
|  | **SW480** | 2735942±363757 | 2565349±286674 | 11271759±6474235 | 6313961±3336391 | 9359286±4889866 |
| **Coherency (a.u.)** | **HT29** | 0,017±0,009 | 0,030±0,021 | 0,024±0,008 | 0,018±0,006 | 0,035±0,034 |
|  | **HCT116** | 0,020±0,011 | 0,040±0,025 | 0,044±0,016 | 0,013±0,005 | 0,013±0,005 |
|  | **SW480** | 0,036±0,017 | 0,021±0,019 | 0,123±0,058 | 0,102±0,046 | 0,077±0,042 |

**Table S6.** Quantitative parameters of SHG signal in co-cultures of colorectal cancer cells and normal fibroblasts in a collagen-based model. Mean±SEM. p-values for statistically significant differences with corresponding monocultures of cancer cells or NFs are shown in the parenthesis.

| **Parameter** | **Co-culture** | **Days of co-cultivation** | | | |
| --- | --- | --- | --- | --- | --- |
|  |  | **1** | **2** | **3** | **7** |
| **Mean(a.u.)** | **NF+HT29** | 3256,9±654,5 | 3531,1±486,0 (<0,00001 vs HT29; 0,00195 vs NF) | 2280,2±332,1 (0,00494 vs NF) | 3400±878,1 (<0,00001 vs HT29; 0,00006 vs NF) |
|  | **NF+HCT116** | 2881±453,3 (0,02066 vs HCT116; 0,02622 vs NF) | 4384,8±924,5 (<0,00001 vs HCT116) | 4092,3±911,8 | 2766,5±571,7 (0,00044 vs HCT116; <0,00001 vs NF) |
|  | **NF+SW480** | 4163,3±489,9 (<0,00001 vs SW480; 0,02690 vs NF) | 3156,2±775, (<0,00001 vs SW480) | 3878,6±590 (0,01154 vs NF) | 3923,6±772,3 (0,00008 vs SW480; 0,00097 vs NF) |
| **Median (a.u.)** | **NF+HT29** | 2839,6±813,4 | 3223,2±410,9 (<0,00001 vs HT29; 0,04251 vs NF) | 1912,5±278,4 (0,01670 vs NF) | 2798±780,8 (<0,00001 vs HT29; 0,00938 vs NF) |
|  | **NF+HCT116** | 2704,8±410 (0,01371 vs NF) | 4177,5±916,6 (<0,00001 vs HCT116) | 3850,5±880,2 | 2453,1±536,4 (0,00169 vs HCT116; 0,00132 vs NF) |
|  | **NF+SW480** | 3722,6±799,3 (0,00017 vs SW480; 0,02597 vs NF) | 2798,3±697,8 (0,00037 vs SW480) | 3132,1±457,6 (0,00037 vs SW480) | 3116,8±713,8 (<0,00001 vs SW480) |
| **Density (a.u.)** | **NF+HT29** | 4357,1±835,1 | 5093,2±597,4 (<0,00001 vs HT29; 0,00153 vs NF) | 3370,6±490,9 (0,00537 vs NF) | 4945,4±1277,1 (<0,00001 vs HT29; 0,00013 vs NF) |
|  | **NF+HCT116** | 4445,2±695,7 (0,02262 vs HCT116) | 6281±1189 (<0,00001 vs HCT116) | 6049,2±1347,9 | 4024±831,5 (0,00022 vs HCT116; <0,00001 vs NF) |
|  | **NF+SW480** | 6209,6±776,9 (0,00001 vs SW480; 0,02398 vs NF) | 4544,2,4±1048,0 (0,00004 vs SW480) | 5641,6±858,2 (0,00476 vs SW480) | 5707±1123,5 (0,00004 vs SW480) |
| **Skew (a.u.)** | **NF+HT29** | 2,07±0,69 (0,00209 vs HT29; 0,03801 vs NF) | 1,94±0,49 | 2,83±0,83 (0,00004 vs HT29) | 2,23±0,34 (0,00733 vs HT29; 0,00249 vs NF) |
|  | **NF+HCT116** | 1,56±0,54 (0,00459 vs NF) | 1,55±0,41 (0,00093 vs NF) | 1,43±0,2 (0,00113 vs NF) | 2,03±0,35 (0,00109 vs HCT116) |
|  | **NF+SW480** | 1,45±0,26 (0,00091 vs SW480; <0,00001 vs NF) | 2,32±0,25 (0,00022 vs NF) | 2,56±0,55 | 2,29±0,42 (0,01072 vs SW480) |
| **Kurtosis (a.u.)** | **NF+HT29** | 9,77±5,11 (0,00372 vs HT29) | 9,37±3,44 | 14,17±6,72 (0,00041 vs HT29) | 8,88±2,81 |
|  | **NF+HCT116** | 6,19±2,58 (0,00074 vs NF) | 6,37±2,77 (0,04013 vs NF) | 5,14±1,99 (0,01944 vs NF) | 7,71±2,95 (0,00014 vs HCT116) |
|  | **NF+SW480** | 5,11±1,82 (0,00163 vs SW480; 0,00001 vs NF) | 11,25±3,75 (0,00391 vs NF) | 11,19±4,88 | 8,52±2,95 (0,01954 vs SW480) |
| **Non-**  **uniformity (a.u.)** | **NF+HT29** | 0,374±0,058 (0,00621 vs HT29; <0,00001 vs NF) | 0,444±0,083 (0,00406 vs HT29; 0,00017) | 0,644±0,159 (0,01736 vs NF) | 0,631±0,121 (<0,00001 vs NF) |
|  | **NF+HCT116** | 0,393±0,062 (0,00379 vs HCT116; <0,00001 vs NF) | 0,347±0,061 (0,00004 vs HCT116; <0,00001 vs NF) | 0,382±0,041 (0,00613 vs HCT116; 0,00006 vs NF) | 0,506±0,061 (<0,00001 vs NF) |
|  | **NF+SW480** | 0,344±0,05 (<0,00001 vs SW480; <0,00001 vs NF) | 0,469±0,083 (<0,00001 vs SW480; <0,00001 vs NF) | 0,674±0,098 (0,00031 vs SW480; 0,02292 vs NF) | 0,707±0,129 (0,00001 vs SW480; 0,00001 vs NF) |
| **Energy (a.u.)** | **NF+HT29** | 4153307±1698421 (<0,00001 vs NF) | 5014442±2138620 (0,00109 vs HT29; <0,00001 vs NF) | 3063164±826500 (0,00048 vs NF) | 6598401±1858260 (<0,00001 vs HT29; <0,00001 vs NF) |
|  | **NF+HCT116** | 2438878±651604 (<0,00001 vs NF) | 4143720±1622357 (0,00023 vs HCT116; <0,00001 vs NF) | 4703123±1611317 (0,00097 vs NF) | 3520102±1145140 (0,01012 vs HCT116; <0,00001 vs NF) |
|  | **NF+SW480** | 4123979±1042188 (0,02088 vs SW480; <0,00001 vs NF) | 4119969±1236084 (0,00006 vs SW480; <0,00001 vs NF) | 10823597±2850149 (0,03931 vs NF) | 7278230±1763705 (<0,00001 vs NF) |
| **Coherency (a.u.)** | **NF+HT29** | 0,084±0,045 (0,00377 vs HT29) | 0,100±0,05 (0,00186 vs HT29) | 0,108±0,044 (0,00034 vs HT29) | 0,129±0,042 (<0,00001 vs HT29) |
|  | **NF+HCT116** | 0,032±0,019 (0,00011 vs NF) | 0,077±0,036 (0,00061 vs HCT116) | 0,093±0,013 (0,00002 vs HCT116) | 0,116±0,036 (0,00006 vs HCT116; 0,03930 vs NF) |
|  | **NF+SW480** | 0,064±0,027 (0,00611 vs NF) | 0,100±0,029 (<0,00001 vs SW480) | 0,159±0,05 | 0,150±0,047 (0,03275 vs SW480) |

**Table S7.** Quantitative parameters of SHG signal in monocultures of normal fibroblasts (NF) and patient-derived CAFs in a collagen-based model. Mean±SEM. p values for statistically significant differences with CAFs are shown in parenthesis.

| **Parameter** | **Cell culture** | **Days of cultivation** | | | | |
| --- | --- | --- | --- | --- | --- | --- |
|  |  | **1** | **2** | **3** | **7** | **9** |
| **Mean (a.u.)** | NF | 3560,8±647 (0,00356) | 4748,6±1144,6 (0,01071) | 4496,9±1374,8 (0,00163) | 5521,4±1471,3 (<0,00001) | 5107,3±1069 (0,00001) |
|  | CAFs | 2779,5±686,5 | 3446,2±951,5 | 1899,2±685,5 | 1589,5±430,9 | 2566±777,8 |
| **Median (a.u.)** | NF | 2835±485,6 (0,0001) | 3946,9±1150,5 | 3512,7±1294,8 (0,00364) | 3859,5±1314 (<0,00001) | 3661,8±954,6 (0,00051) |
|  | CAFs | 1976,9±535,8 | 3197,9±946,8 | 1369,9±604,2 | 938,9±158,3 | 1996,2±806 |
| **Density (a.u.)** | NF | 5175,3±940,4 (0,03348) | 6901,6±1663,6 (0,01071) | 6535,7±1998,1 (0,00163) | 7701±2052,1 (<0,00001) | 7123,4±1491 (0,00001) |
|  | CAFs | 4343±1072,7 | 5008,8±1382,9 | 2760,3±996,2 | 2216,9±601 | 3579±1084,8 |
| **Skew (a.u.)** | NF | 2,72±0,49 (0,01688) | 2,22±0,52 (0,00196) | 2,31±0,44 (0,0392) | 2,32±0,54 (0,0083) | 2,13±0,35 |
|  | CAFs | 3,16±0,47 | 1,45±0,44 | 3,35±1,13 | 3,77±1,13 | 2,91±1,17 |
| **Kurtosis (a.u.)** | NF | 13,58±5,13 | 9,04±3,66 | 9,32±3,45 (0,02941) | 8,24±3,97 (0,00063) | 6,90±2,43 (0,0009) |
|  | CAFs | 16,75±6,17 | 5,57±3,71 | 23,50±14,65 | 20,14±6,26 | 16,01±5,55 |
| **Non-**  **Uniformity (a.u.)** | NF | 0,77±0,092 (0,00007) | 0,692±0,178 (0,00193) | 0,827±0,133 (0,03904) | 0,988±0,162 (0,00009) | 0,919±0,102 |
|  | CAFs | 0,994±0,146 | 0,497±0,073 | 1,082±0,27 | 1,261±0,105 | 0,877±0,204 |
| **­­** | NF | 10869275±2985087 | 16075222±4299296 (<0,00001) | 17015339±7448400 (0,00111) | 31620550±11366406 (<0,00001) | 27640223±11476127 (0,00002) |
|  | CAFs | 12134524±3021789 | 5958785±2150234 | 4953199±1708736 | 4073382±1521828 | 6618530±2158566 |
| **Coherency (a.u.)** | NF | 0,115±0,046 | 0,092±0,039 (0,0066) | 0,118±0,051 | 0,153±0,041 (0,0035) | 0,143±0,068 |
|  | CAFs | 0,119±0,034 | 0,050±0,029 | 0,128±0,020 | 0,094±0,045 | 0,099±0,050 |

**Table S8.** List of ECM and collagen biosynthesis, collagen positive regulation, and COL genes (B+PR gene category).

| **Official gene symbol** | **Gene name** | **Gene category** | **Source** |
| --- | --- | --- | --- |
| ADAMTS3 | ADAM metallopeptidase with thrombospondin type 1 motif 3 | Protease (protocollagen aminopropeptidase) | GO:0032964 |
| AMELX | amelogenin, X-linked | ECM component | GO:0032964; GO:0032967 |
| ARG1 | arginase 1 | Aminoacis metabolism enzyme | GO:0032964 |
| ARRB2 | arrestin beta 2 | Cellular signaling-involved protein (G-protein-coupled receptor desensitization) | GO:0032964; GO:0032967 |
| BMP4 | bone morphogenetic protein 4 | Cellular signaling-involved protein (TGF-beta signaling activation) | GO:0032964; GO:0032967 |
| CBX8 | chromobox 8 | Component of Polycomb group multiprotein complex PRC-1 | GO:0032964; GO:0032967 |
| CCN2 | cellular communication network factor 2 | Cellular signaling-involved protein (TGF-beta signaling) | GO:0032964; GO:0032967 |
| COL10A1 | collagen type X alpha 1 chain | ECM component | COL gene |
| COL11A1 | collagen type XI alpha 1 chain | ECM component | COL gene |
| COL11A2 | collagen type XI alpha 2 chain | ECM component | COL gene |
| COL12A1 | collagen type XII alpha 1 chain | ECM component | COL gene |
| COL13A1 | collagen type XIII alpha 1 chain | ECM component | COL gene |
| COL14A1 | collagen type XIV alpha 1 chain | ECM component | COL gene |
| COL15A1 | collagen type XV alpha 1 chain | ECM component | COL gene |
| COL16A1 | collagen type XVI alpha 1 chain | ECM component | COL gene |
| COL17A1 | collagen type XVII alpha 1 chain | ECM component | COL gene |
| COL18A1 | collagen type XVIII alpha 1 chain | ECM component | COL gene |
| COL19A1 | collagen type XIX alpha 1 chain | ECM component | COL gene |
| COL1A1 | collagen type I alpha 1 chain | ECM component | GO:0032964; COL gene |
| COL1A2 | collagen type I alpha 2 chain | ECM component | COL gene |
| COL20A1 | collagen type XX alpha 1 chain | ECM component | COL gene |
| COL21A1 | collagen type XXI alpha 1 chain | ECM component | COL gene |
| COL22A1 | collagen type XXII alpha 1 chain | ECM component | COL gene |
| COL23A1 | collagen type XXIII alpha 1 chain | ECM component | COL gene |
| COL24A1 | collagen type XXIV alpha 1 chain | ECM component | COL gene |
| COL25A1 | collagen type XXV alpha 1 chain | ECM component | COL gene |
| COL26A1 | collagen type XXVI alpha 1 chain | ECM component | COL gene |
| COL27A1 | collagen type XXVII alpha 1 chain | ECM component | COL gene |
| COL28A1 | collagen type XXVIII alpha 1 chain | ECM component | COL gene |
| COL2A1 | collagen type II alpha 1 chain | ECM component | COL gene |
| COL3A1 | collagen type III alpha 1 chain | ECM component | COL gene |
| COL4A1 | collagen type IV alpha 1 chain | ECM component | COL gene |
| COL4A2 | collagen type IV alpha 2 chain | ECM component | COL gene |
| COL4A3 | collagen type IV alpha 3 chain | ECM component | COL gene |
| COL4A4 | collagen type IV alpha 4 chain | ECM component | COL gene |
| COL4A5 | collagen type IV alpha 5 chain | ECM component | COL gene |
| COL4A6 | collagen type IV alpha 6 chain | ECM component | COL gene |
| COL5A1 | collagen type V alpha 1 chain | ECM component | GO:0032964; COL gene |
| COL5A2 | collagen type V alpha 2 chain | ECM component | COL gene |
| COL5A3 | collagen type V alpha 3 chain | ECM component | COL gene |
| COL6A1 | collagen type VI alpha 1 chain | ECM component | COL gene |
| COL6A2 | collagen type VI alpha 2 chain | ECM component | COL gene |
| COL6A3 | collagen type VI alpha 3 chain | ECM component | COL gene |
| COL6A5 | collagen type VI alpha 5 chain | ECM component | COL gene |
| COL6A6 | collagen type VI alpha 6 chain | ECM component | COL gene |
| COL7A1 | collagen type VII alpha 1 chain | ECM component | COL gene |
| COL8A1 | collagen type VIII alpha 1 chain | ECM component | COL gene |
| COL8A2 | collagen type VIII alpha 2 chain | ECM component | COL gene |
| COL9A1 | collagen type IX alpha 1 chain | ECM component | COL gene |
| COL9A2 | collagen type IX alpha 2 chain | ECM component | COL gene |
| COL9A3 | collagen type IX alpha 3 chain | ECM component | COL gene |
| COLGALT1 | collagen beta(1-O)galactosyltransferase 1 | Collagen processing-involved protein | GO:1904028 |
| COLGALT2 | collagen beta(1-O)galactosyltransferase 2 | Collagen processing-involved protein | COLGALT1 paralog |
| CREB3L1 | cAMP responsive element binding protein 3 like 1 | Transcription factor | GO:0032964; GO:0032967 |
| ELN | elastin | ECM component | GO:0005201 |
| ENG | endoglin | Cellular signaling-involved protein (TGF-beta signaling ) | GO:0032964; GO:0032967 |
| F2 | coagulation factor II, thrombin | Coagulation factor | GO:0032964; GO:0032967 |
| F2R | coagulation factor II thrombin receptor | Coagulation factor receptor | GO:0032964; GO:0032967 |
| FN1 | fibronectin 1 | ECM component | GO:0005201 |
| HDAC2 | histone deacetylase 2 | Transcription corepressor | GO:0032964; GO:0032967 |
| IHH | indian hedgehog | Cellular signaling-involved protein (hedgehog signaling ) | GO:0032964; GO:0032967 |
| ITGA2 | integrin subunit alpha 2 | Integrin receptor part | GO:0032964; GO:0032967 |
| LAMB1 | laminin subunit beta 1 | ECM component | GO:0005201 |
| LAMB2 | laminin subunit beta 2 | ECM component | GO:0005201 |
| LAMB3 | laminin subunit beta 3 | ECM component | GO:0005201 |
| LARP6 | La ribonucleoprotein domain family member 6 | Collagen translation regulator | GO:0032964; GO:0032967 |
| MYB | MYB proto-oncogene, transcription factor | Transcription factor | GO:0032964; GO:0032967 |
| P3H3 | prolyl 3-hydroxylase 3 | Collagen processing-involved protein | GO:0032964 |
| P3H4 | prolyl 3-hydroxylase family member 4 (non-enzymatic) | Collagen processing-involved protein | GO:0032964 |
| PDGFRB | platelet derived growth factor receptor beta | Receptor tyrosine kinase | GO:0032964; GO:0032967 |
| RCN3 | reticulocalbin 3 | Collagen secretion regulator | GO:0032964 |
| RGCC | regulator of cell cycle | Cell cycle regulator | GO:0032964; GO:0032967 |
| SCX | scleraxis bHLH transcription factor | Transcription factor | GO:0032964; GO:0032967 |
| SERPINB7 | serpin family B member 7 | Protease inhibitor | GO:0032964; GO:0032967 |
| SERPINF2 | serpin family F member 2 | Protease inhibitor | GO:0032964; GO:0032967 |
| SERPINH1 | serpin family H member 1 | Protease inhibitor | GO:0032964 |
| SUCO | SUN domain containing ossification factor | Collagen biosynthesis regulator | GO:0032964; GO:0032967 |
| TGFB1 | transforming growth factor beta 1 | Cellular signaling-involved protein (TGF-beta signaling ) | GO:0032964; GO:0032967 |
| TGFB3 | transforming growth factor beta 3 | Cellular signaling-involved protein (TGF-beta signaling ) | GO:0032964; GO:0032967 |
| TNC | tenascin C | ECM component | GO:0005201 |
| TRAM2 | translocation associated membrane protein 2 | Posttranslational protein processing | GO:0032964 |
| UCN | urocortin | Hormone | GO:0032964; GO:0032967 |
| VIM | vimentin | Cytoskeleton protein | GO:0032964; GO:0032967 |
| VSIR | V-Set Immunoregulatory Receptor | Multifunctional protein | GO:0032967 |
| WNT4 | Wnt family member 4 | Cellular signaling-involved protein (Wnt signaling ) | GO:0032964; GO:0032967 |

**Table S9.** List of collagen fibril organization and ECM aggregation related genes (CFO+EA gene category).

| **Official gene symbol** | **Gene name** | **Gene category** | **Source** |
| --- | --- | --- | --- |
| AEBP1 | AE binding protein 1 | Positive regulator of collagen fibrillogenesis | GO:0030199 |
| ANXA2 | annexin A2 | Pleiotropic protein | GO:0030199 |
| ATP7A | ATPase copper transporting alpha | Copper transporter | GO:0030199 |
| BGN | biglycan | ECM component | GO:0030198 |
| CYP1B1 | cytochrome P450 family 1 subfamily B member 1 | Cytochrome P450 | GO:0030199 |
| DPT | dermatopontin | ECM component | GO:0030199 |
| FMOD | fibromodulin | ECM component | GO:0030199 |
| GREM1 | gremlin 1, DAN family BMP antagonist | Cellular signaling-involved protein (inhibition of TGF-beta and Wnt signaling) | GO:0030199 |
| LOX | lysyl oxidase | ECM processing enzyme | GO:0030199 |
| LOXL1 | lysyl oxidase like 1 | ECM processing enzyme | GO:0030198 |
| LOXL2 | lysyl oxidase like 2 | ECM processing enzyme | GO:0030199 |
| LOXL3 | lysyl oxidase like 3 | ECM processing enzyme | GO:0030199 |
| LOXL4 | lysyl oxidase like 4 | ECM processing enzyme | GO:0030199 |
| LUM | lumican | ECM component | GO:0030199 |
| NF1 | neurofibromin 1 | Cellular signaling-involved protein (inhibition of Ras signaling) | GO:0030199 |
| P4HA1 | prolyl 4-hydroxylase subunit alpha 1 | ECM processing enzyme | GO:0030199 |
| PLOD1 | procollagen-lysine,2-oxoglutarate 5-dioxygenase 1 | ECM processing enzyme | PLOD3 paralog |
| PLOD2 | procollagen-lysine,2-oxoglutarate 5-dioxygenase 2 | ECM processing enzyme | PLOD3 paralog |
| PLOD3 | procollagen-lysine,2-oxoglutarate 5-dioxygenase 3 | ECM processing enzyme | GO:0030199 |
| PXDN | peroxidasin | ECM processing-involved protein | GO:0030199 |
| RB1 | RB transcriptional corepressor 1 | Transcriptional corepressor | GO:0030199 |
| TGFB2 | transforming growth factor beta 2 | Cellular signaling-involved protein (TGF-beta signaling) | GO:0030199 |
| TGFBR1 | transforming growth factor beta receptor 1 | Cellular signaling-involved protein (TGF-beta signaling) | GO:0030199 |
| TNXB | tenascin XB | ECM component | GO:0030199 |
| VIPAS39 | VPS33B interacting protein, apical-basolateral polarity regulator, spe-39 homolog | Multifunctional protein | GO:0030199 |

**Table S10.** List of collagen catabolism and negative regulation related genes (C+NR gene category).

| **Official gene symbol** | **Gene name** | | **Gene category** | **Source** | |
| --- | --- | --- | --- | --- | --- |
| ADAM15 | ADAM metallopeptidase domain 15 | | Protease (metallopeptidase) | GO:0030574 | |
| ADAMTS14 | ADAM metallopeptidase with thrombospondin type 1 motif 14 | | Protease (metallopeptidase) | GO:0030574 | |
| ADAMTS2 | ADAM metallopeptidase with thrombospondin type 1 motif 2 | | Protease (metallopeptidase) | GO:0030574 | |
| ADAMTS3 | ADAM metallopeptidase with thrombospondin type 1 motif 3 | | Protease (metallopeptidase) | GO:0030574 | |
| CIITA | class II major histocompatibility complex transactivator | | Transcription coactivator | GO:0032966 | |
| COL13A1 | collagen type XIII alpha 1 chain | | ECM component | GO:0030574 | |
| COL15A1 | collagen type XV alpha 1 chain | | ECM component (basal membrane) | GO:0030574 | |
| COL19A1 | collagen type XIX alpha 1 chain | | ECM component | GO:0030574 | |
| CST3 | cystatin C | | Protease inhibitor | GO:0030574;  GO:0032966 | |
| CTSB | cathepsin B | | Protease | GO:0030574 | |
| CTSD | cathepsin D | | Protease | GO:0030574 | |
| CTSK | cathepsin K | | Protease | GO:0030574 | |
| CTSL | cathepsin L | | Protease | GO:0030574 | |
| CTSS | cathepsin S | | Protease | GO:0030574 | |
| CYGB | cytoglobin | | Globin protein | GO:0032966 | |
| EMILIN1 | elastin microfibril interfacer 1 | | ECM component | GO:0032966 | |
| ERRFI1 | ERBB receptor feedback inhibitor 1 | | Cellular signaling-involved protein | GO:0032966 | |
| FAP | fibroblast activation protein alpha | | Protease (integral membrane gelatinase) | GO:0030574 | |
| FURIN | furin, paired basic amino acid cleaving enzyme | | Protease | GO:0030574 | |
| GOT1 | glutamic-oxaloacetic transaminase 1 | | Aminoacid metabolism enzyme | GO:0032966 | |
| IL6 | interleukin 6 | | Cytokine | GO:0032966 | |
| ITGB1 | integrin subunit beta 1 | | Interleukin | GO:0030574 | |
| KLK6 | kallikrein related peptidase 6 | | Protease | GO:0030574 | |
| MMP1 | matrix metallopeptidase 1 | | Protease (matrix metallopeptidase) | GO:0030574 | |
| MMP10 | matrix metallopeptidase 10 | | Protease (matrix metallopeptidase) | GO:0030574 | |
| MMP11 | matrix metallopeptidase 11 | | Protease (matrix metallopeptidase) | GO:0030574 | |
| MMP12 | matrix metallopeptidase 12 | | Protease (matrix metallopeptidase) | GO:0030574 | |
| MMP13 | matrix metallopeptidase 13 | | Protease (matrix metallopeptidase) | GO:0030574 | |
| MMP14 | matrix metallopeptidase 14 | | Protease (matrix metallopeptidase) | GO:0030574 | |
| MMP15 | matrix metallopeptidase 15 | | Protease (matrix metallopeptidase) | GO:0030574 | |
| MMP16 | matrix metallopeptidase 16 | | Protease (matrix metallopeptidase) | GO:0030574 | |
| MMP17 | matrix metallopeptidase 17 | | Protease (matrix metallopeptidase) | GO:0030574 | |
| MMP19 | matrix metallopeptidase 19 | | Protease (matrix metallopeptidase) | GO:0030574 | |
| MMP2 | matrix metallopeptidase 2 | | Protease (matrix metallopeptidase) | GO:0030574 | |
| MMP20 | matrix metallopeptidase 20 | | Protease (matrix metallopeptidase) | GO:0030574 | |
| MMP21 | matrix metallopeptidase 21 | | Protease (matrix metallopeptidase) | GO:0030574 | |
| MMP23B | matrix metallopeptidase 23B | | Protease (matrix metallopeptidase) | GO:0030574 | |
| MMP24 | matrix metallopeptidase 24 | | Protease (matrix metallopeptidase) | GO:0030574 | |
| MMP25 | matrix metallopeptidase 25 | | Protease (matrix metallopeptidase) | GO:0030574 | |
| MMP26 | matrix metallopeptidase 26 | | Protease (matrix metallopeptidase) | GO:0030574 | |
| MMP27 | matrix metallopeptidase 27 | | Protease (matrix metallopeptidase) | GO:0030574 | |
| MMP28 | matrix metallopeptidase 28 | | Protease (matrix metallopeptidase) | GO:0030574 | |
| MMP3 | matrix metallopeptidase 3 | | Protease (matrix metallopeptidase) | GO:0030574 | |
| MMP7 | matrix metallopeptidase 7 | | Protease (matrix metallopeptidase) | GO:0030574 | |
| MMP8 | matrix metallopeptidase 8 | | Protease (matrix metallopeptidase) | GO:0030574 | |
| MMP9 | matrix metallopeptidase 9 | | Protease (matrix metallopeptidase) | GO:0030574 | |
| MRC2 | mannose receptor C type 2 | | Mediator of collagen lysosomal degradation | GO:0030574 | |
| NPPC | natriuretic peptide C | | Hormone | GO:0032966 | |
| PEPD | peptidase D | | Protease | GO:0030574 | |
| PHYKPL | 5-phosphohydroxy-L-lysine phospho-lyase | | Aminoacid metabolism enzyme | GO:0030574 | |
| PLAU | plasminogen activator, urokinase | | Protease | [26] | |
| PPARD | peroxisome proliferator activated receptor delta | | Transcription factor | GO:0032966 | |
| PPARG | peroxisome proliferator activated receptor gamma | | Transcription factor | GO:0032966 | |
| PRSS2 | protease, serine 2 | | Protease | GO:0030574 | |
| PRTN3 | protease 3 | | Protease | GO:0030574 | |
| RAP1A | RAP1A, member of RAS oncogene family | | Cellular signaling-involved protein | GO:0032966 | |
| TMPRSS6 | transmembrane protease, serine 6 | | Protease | GO:0030574 | |
| VSIR | V-Set Immunoregulatory Receptor | | Multifunctional protein | GO:0030574 | |
|  | | © 2020 by the authors. Submitted for possible open access publication under the terms and conditions of the Creative Commons Attribution (CC BY) license (http://creativecommons.org/licenses/by/4.0/). | | |  |
